# Supplementary material for: Lipoic/Capsaicin-Related Amides: Synthesis and Biological Characterization of New TRPV1 Agonists Endowed with Protective Properties against Oxidative Stress
Source: Int J Mol Sci. 2022 Nov 5;23(21):13580. doi: 10.3390/ijms232113580 (PMC9658705; doi:10.3390/ijms232113580)
Supplement: Supplementary file 1 [file ijms-23-13580-s001.zip › ijms-2012027-supplementary.pdf]

“Supplementary Materials”

Lipoic/capsaicin-related amides. Synthesis and biological characterization of new TRPV1 agonists endowed with protective properties against oxidative stress

Antonella Brizzi [1,*,†], Samuele Maramai [1,†], Francesca Aiello [2] Maria Camilla Baratto [1], Federico Corelli [1], Claudia Mugnaini [1], Marco Paolino [1], Francesco Scorzelli [3], Carlo Aldinucci [4], Luciano De Petrocellis [5], Cinzia Signorini [4] and Federica Pessina [4,*]

Table of contents

S2-S19 ^{1}H-NMR and ^{13}C-NMR of compounds **1-9**.

S20 Figure S20. Scavenger activity of capsaicin through the DPPH assay using EPR spectroscopy.

S21 Figure S21. Cell viability of capsaicin (caps) and tested lipoic amides **1**, **4-5**, **7** and **9**.

.

Compound **1**

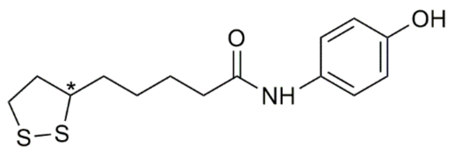

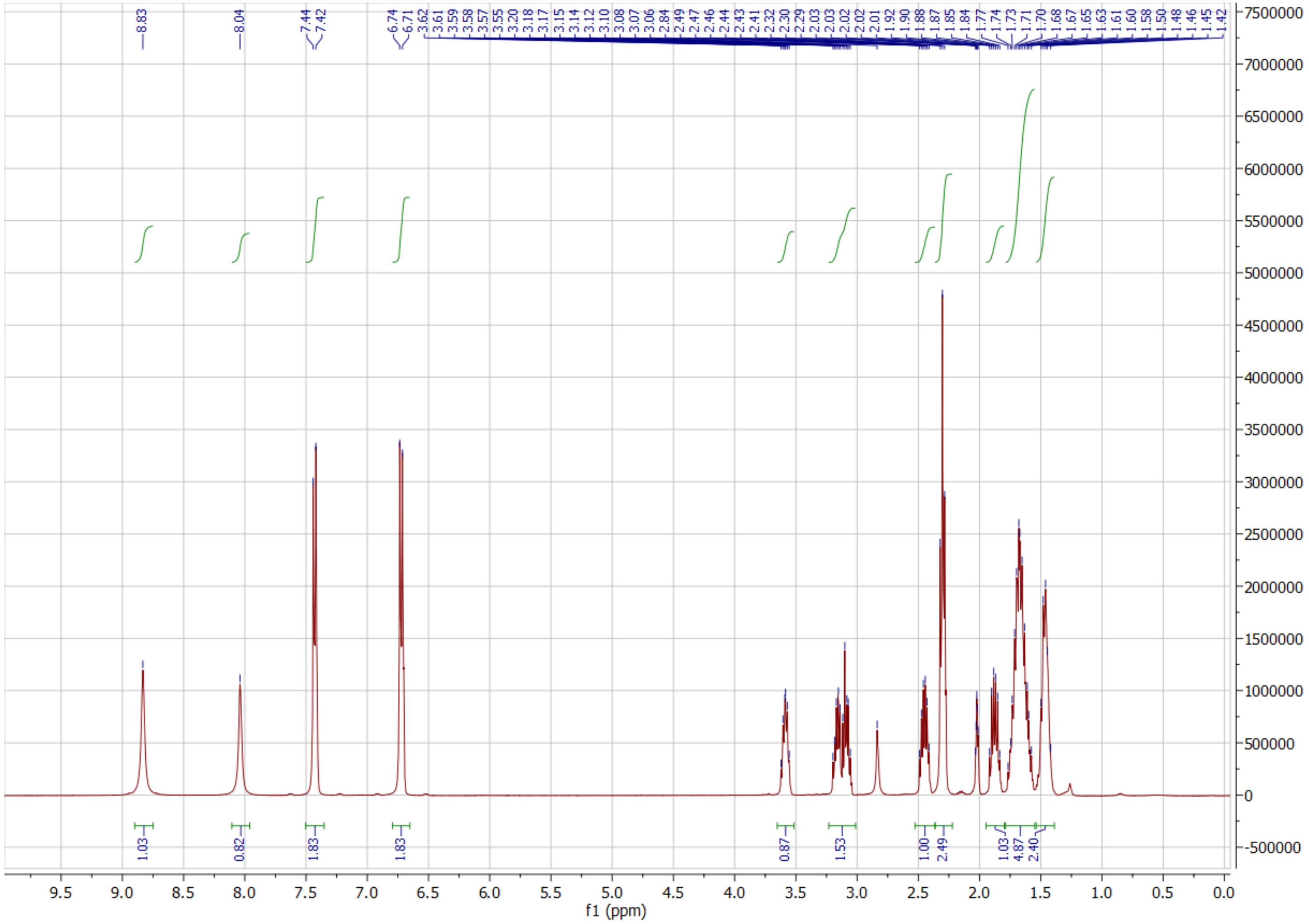

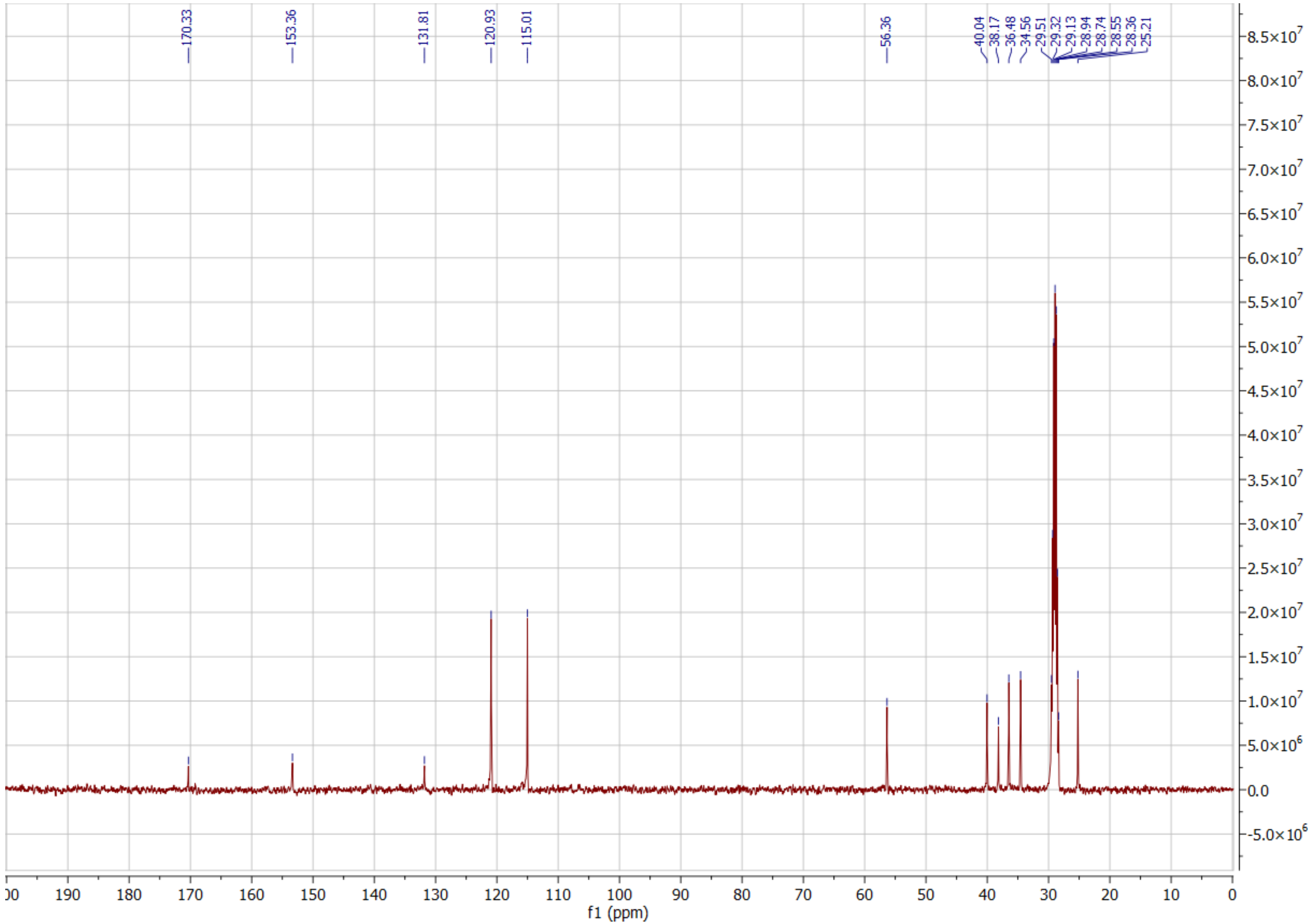
170.33
153.36
131.81
120.93
115.01
56.36
40.04
38.17
36.48
34.56
29.51
29.32
29.13
28.94
28.74
28.55
28.36
25.21
f1 (ppm)

Compound **2**

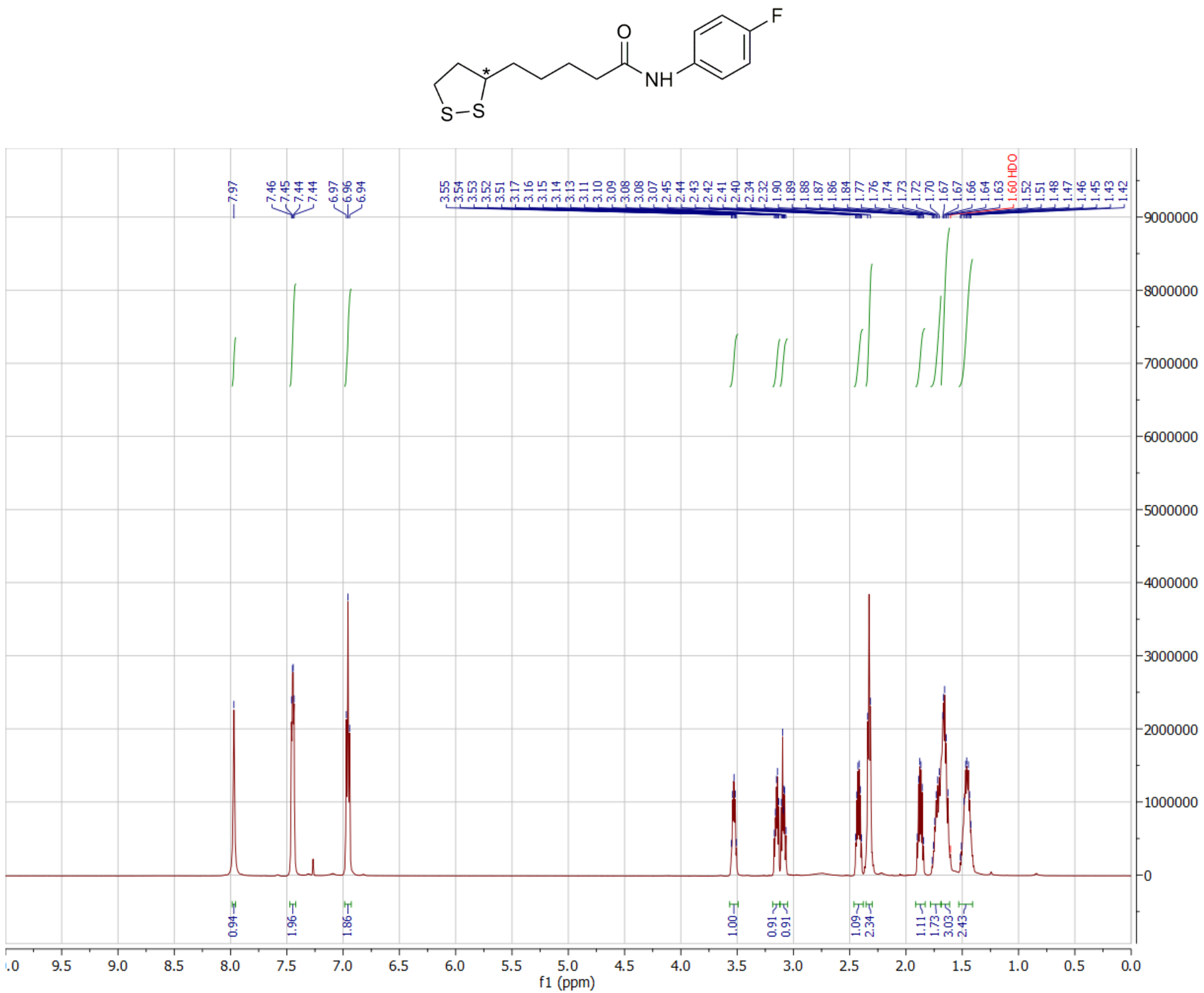

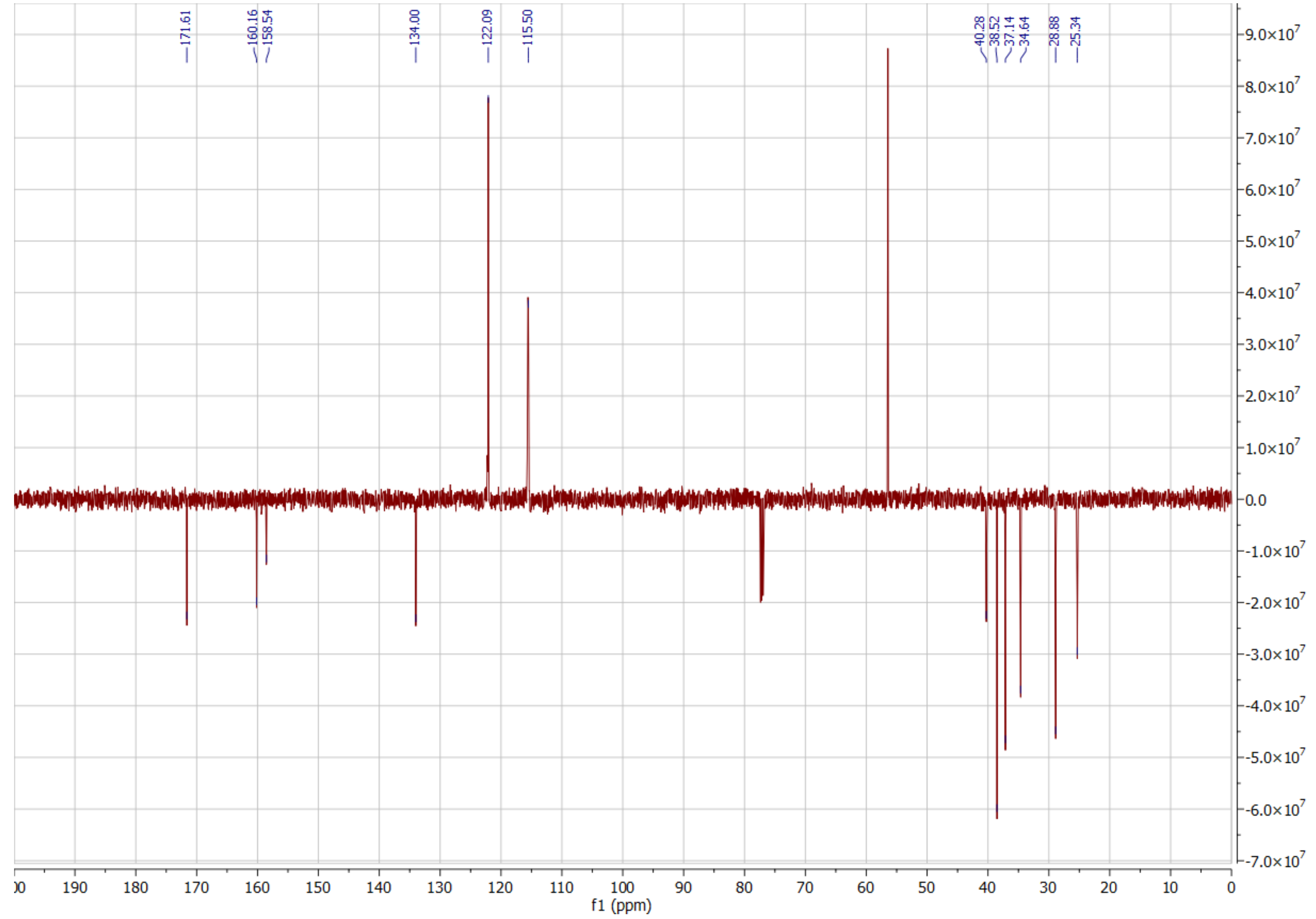

171.61
160.16
158.54
134.00
122.09
115.50
40.28
38.52
37.14
34.64
28.88
25.34
9.0×10^7
8.0×10^7
7.0×10^7
6.0×10^7
5.0×10^7
4.0×10^7
3.0×10^7
2.0×10^7
1.0×10^7
0.0
-1.0×10^7
-2.0×10^7
-3.0×10^7
-4.0×10^7
-5.0×10^7
-6.0×10^7
-7.0×10^7
190 180 170 160 150 140 130 120 110 100 90 80 70 60 50 40 30 20 10 0
f1 (ppm)

Compound **3**

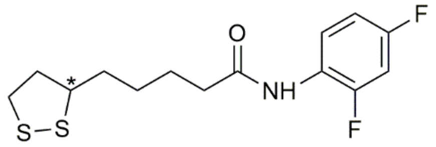

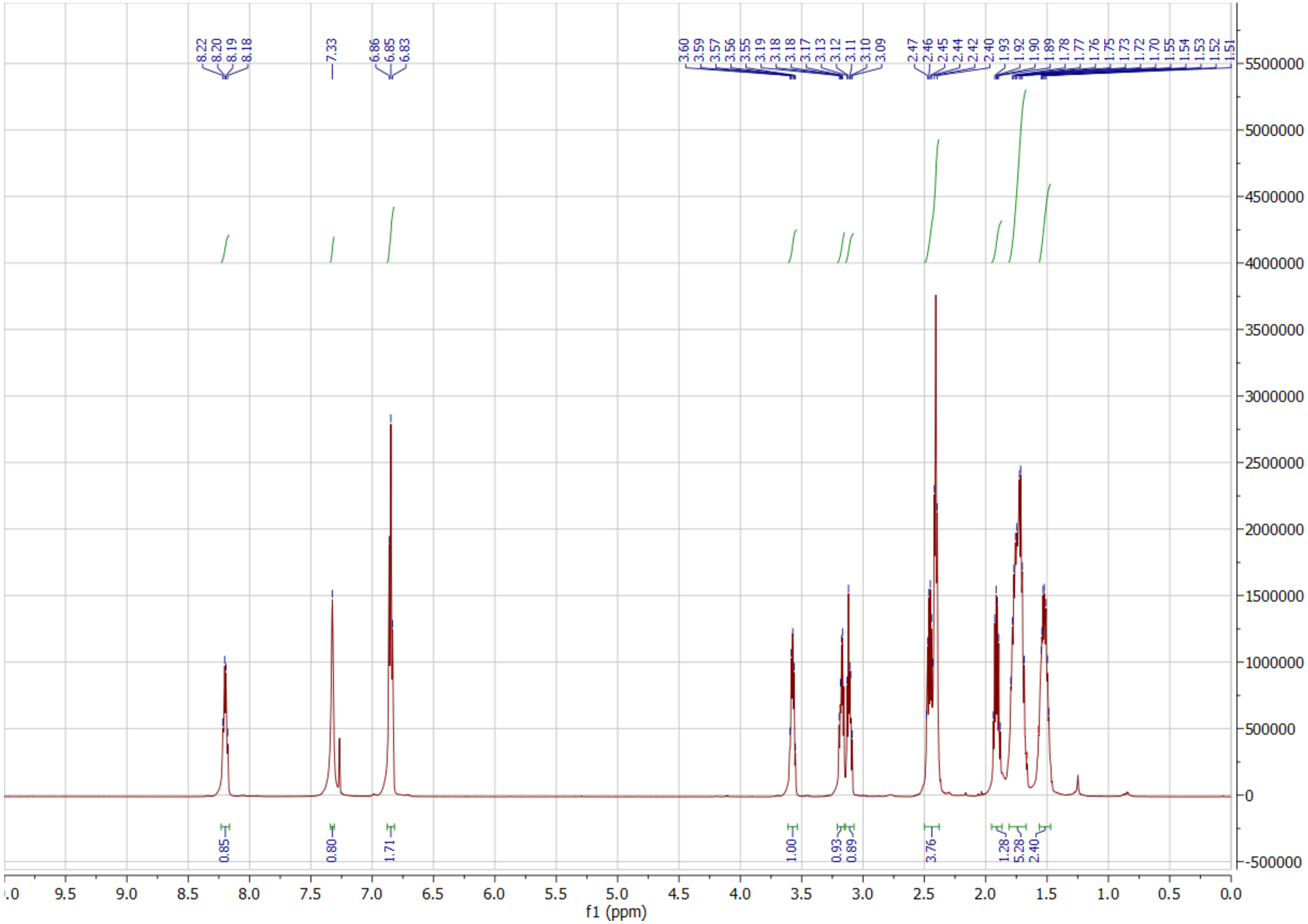
8.22
8.20
8.19
8.18
7.33
6.86
6.85
6.83
3.60
3.59
3.57
3.56
3.55
3.19
3.18
3.18
3.17
3.13
3.12
3.11
3.10
3.09
2.47
2.46
2.45
2.44
2.42
2.40
1.93
1.92
1.90
1.89
1.78
1.77
1.76
1.75
1.73
1.72
1.70
1.55
1.54
1.53
1.52
1.51
0.85
0.80
1.71
1.00
0.93
0.89
3.76
1.28
5.28
2.40
f1 (ppm)

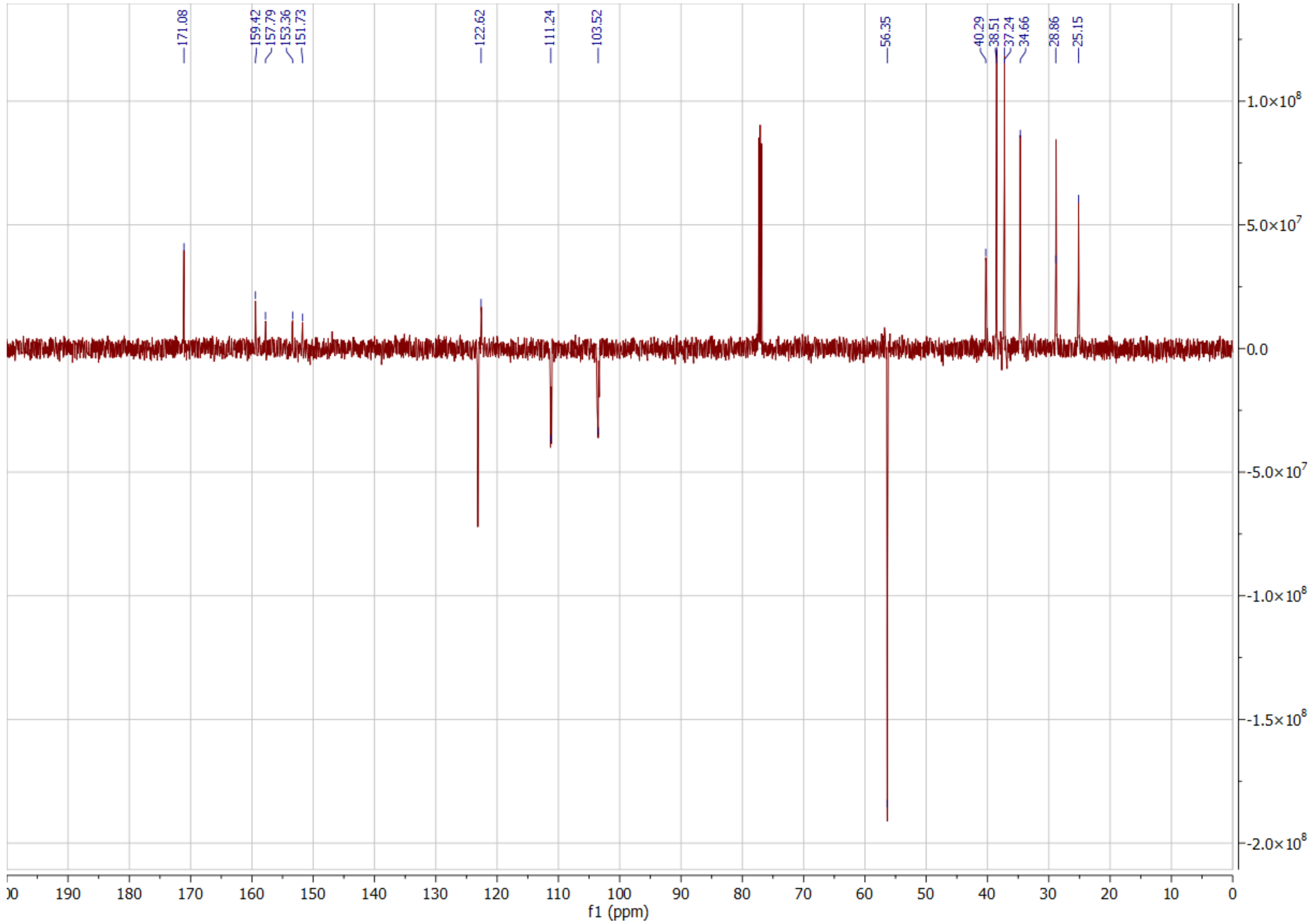
171.08
159.42
157.79
153.36
151.73
122.62
111.24
103.52
56.35
40.29
38.51
37.24
34.66
28.86
25.15
1.0×10^8
5.0×10^7
0.0
-5.0×10^7
-1.0×10^8
-1.5×10^8
-2.0×10^8
190
180
170
160
150
140
130
120
110
100
90
80
70
60
50
40
30
20
10
0
f1 (ppm)

Compound **4**

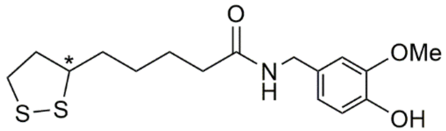

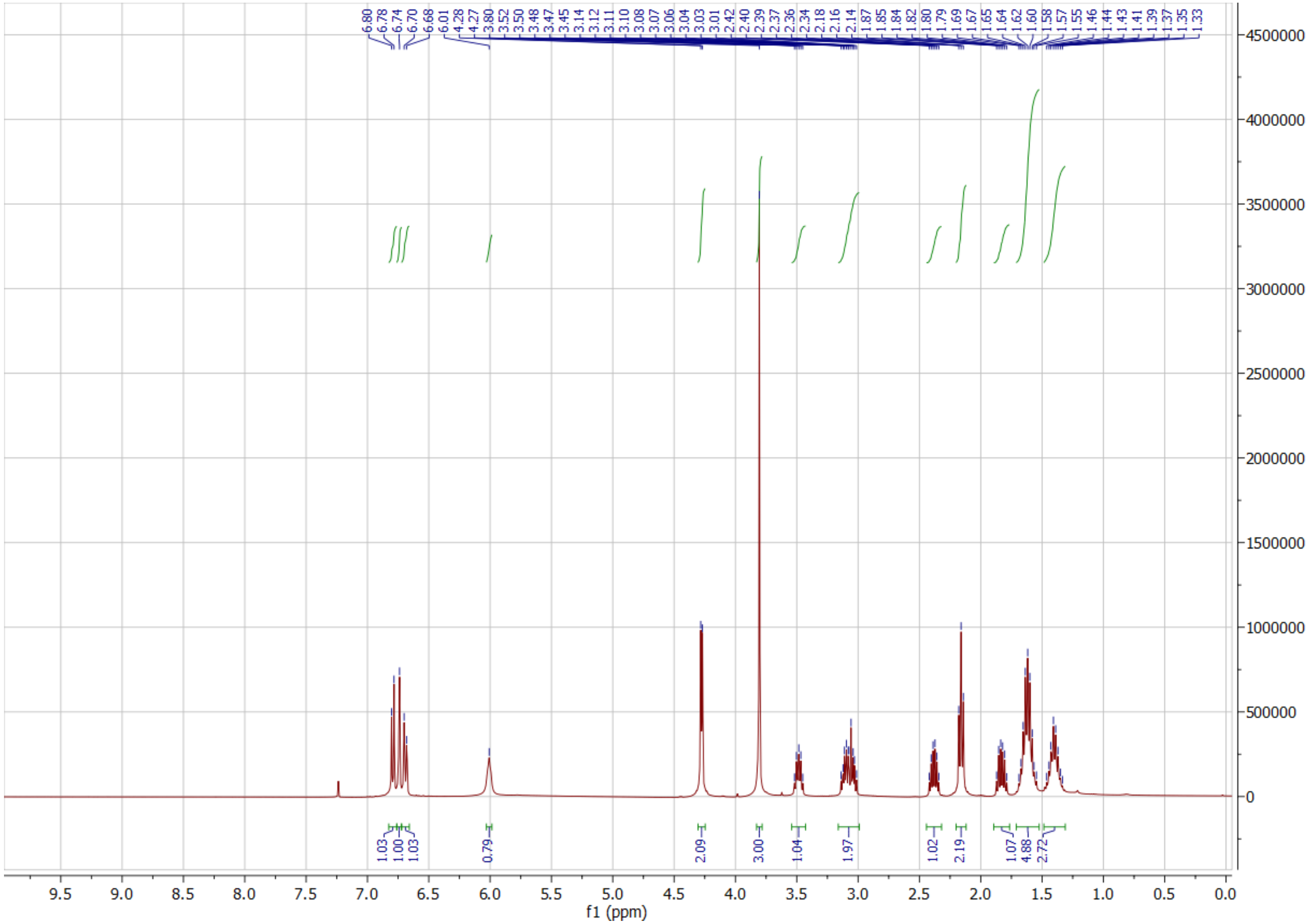

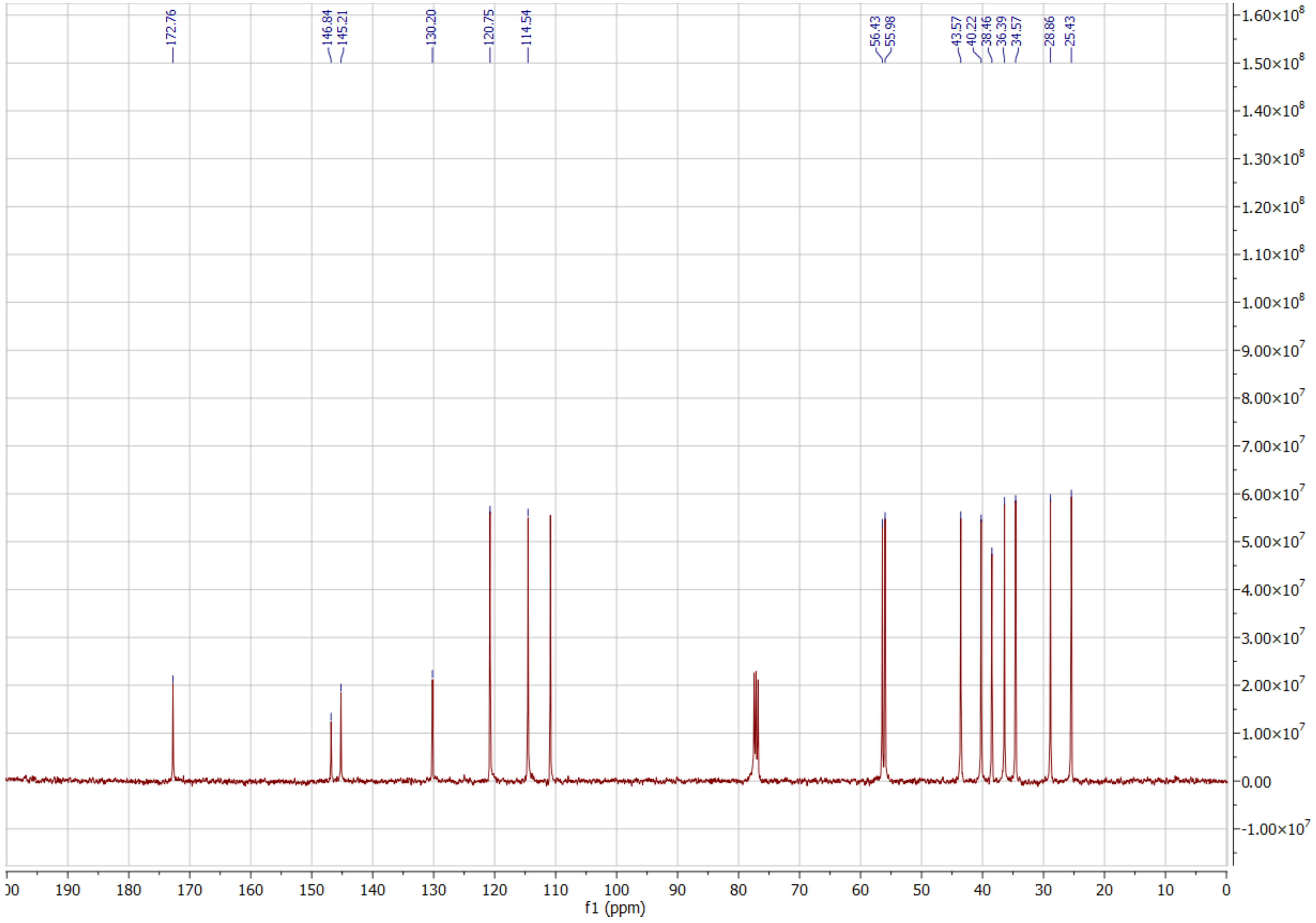
172.76
146.84
145.21
130.20
120.75
114.54
56.43
55.98
43.57
40.22
38.46
36.39
34.57
28.86
25.43
f1 (ppm)

Compound **5**

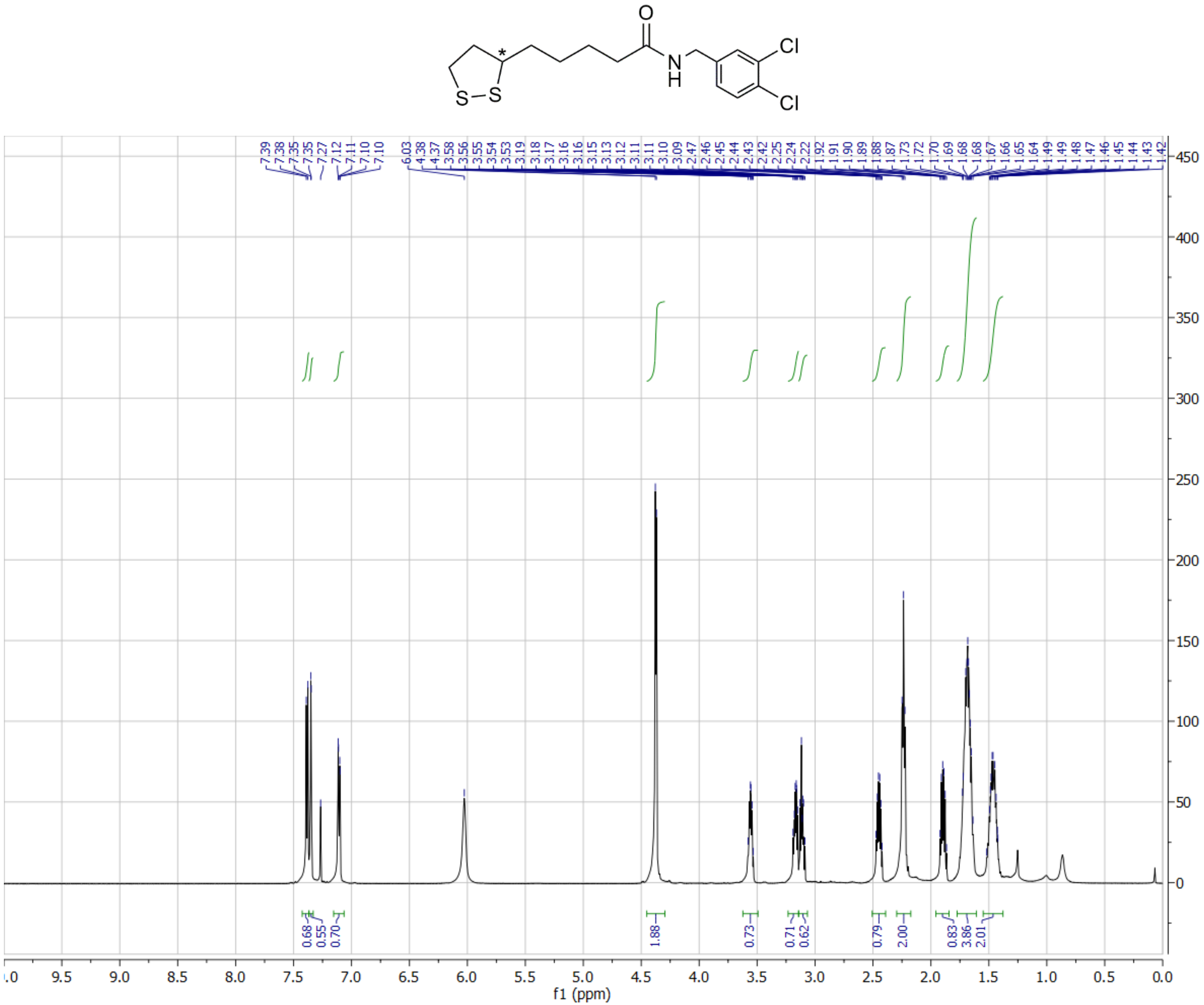

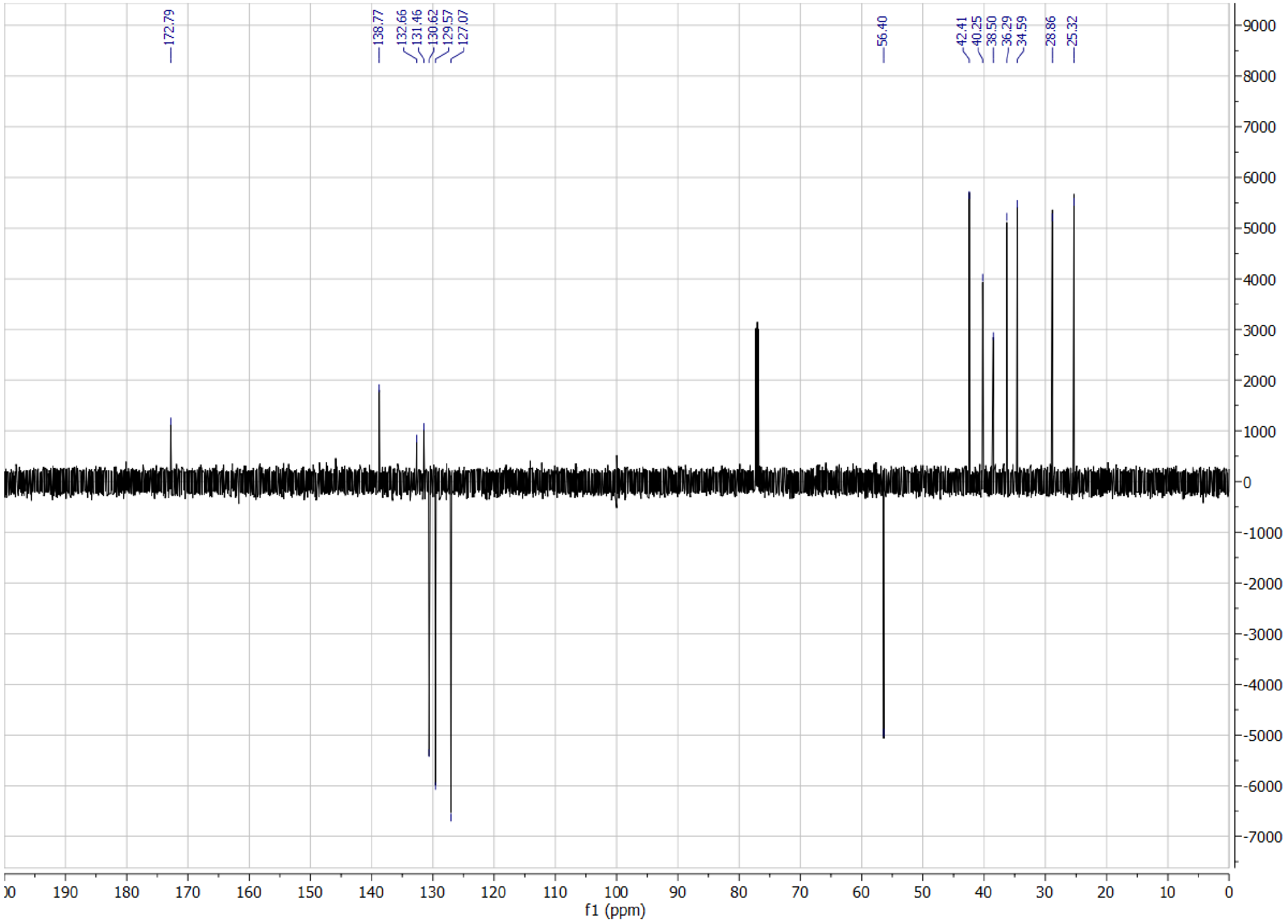
172.79
138.77
132.66
131.46
130.62
129.57
127.07
56.40
42.41
40.25
38.50
36.29
34.59
28.86
25.32
f1 (ppm)

Compound **6**

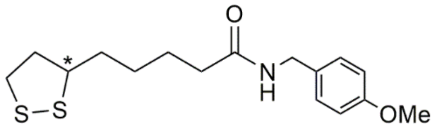

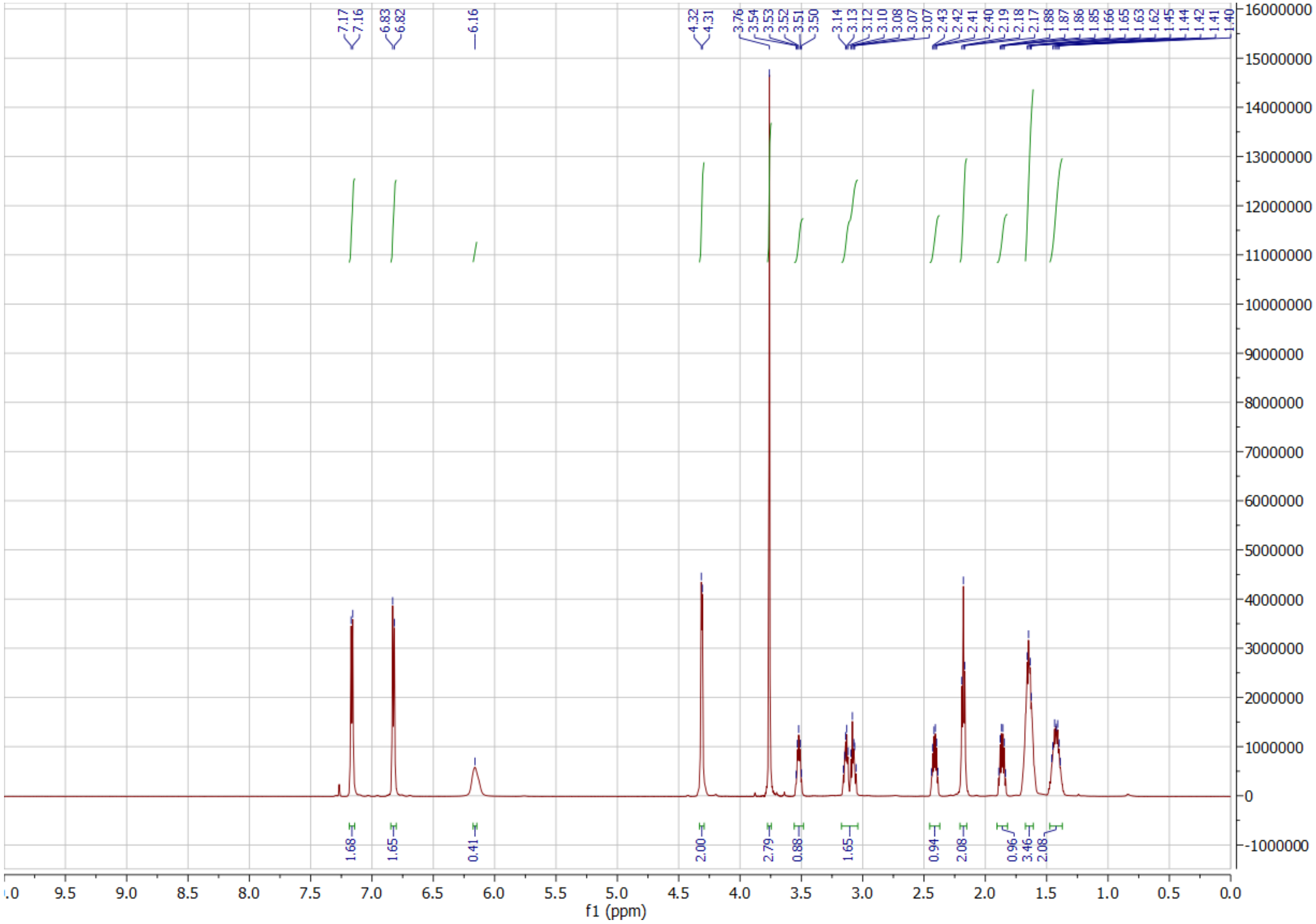

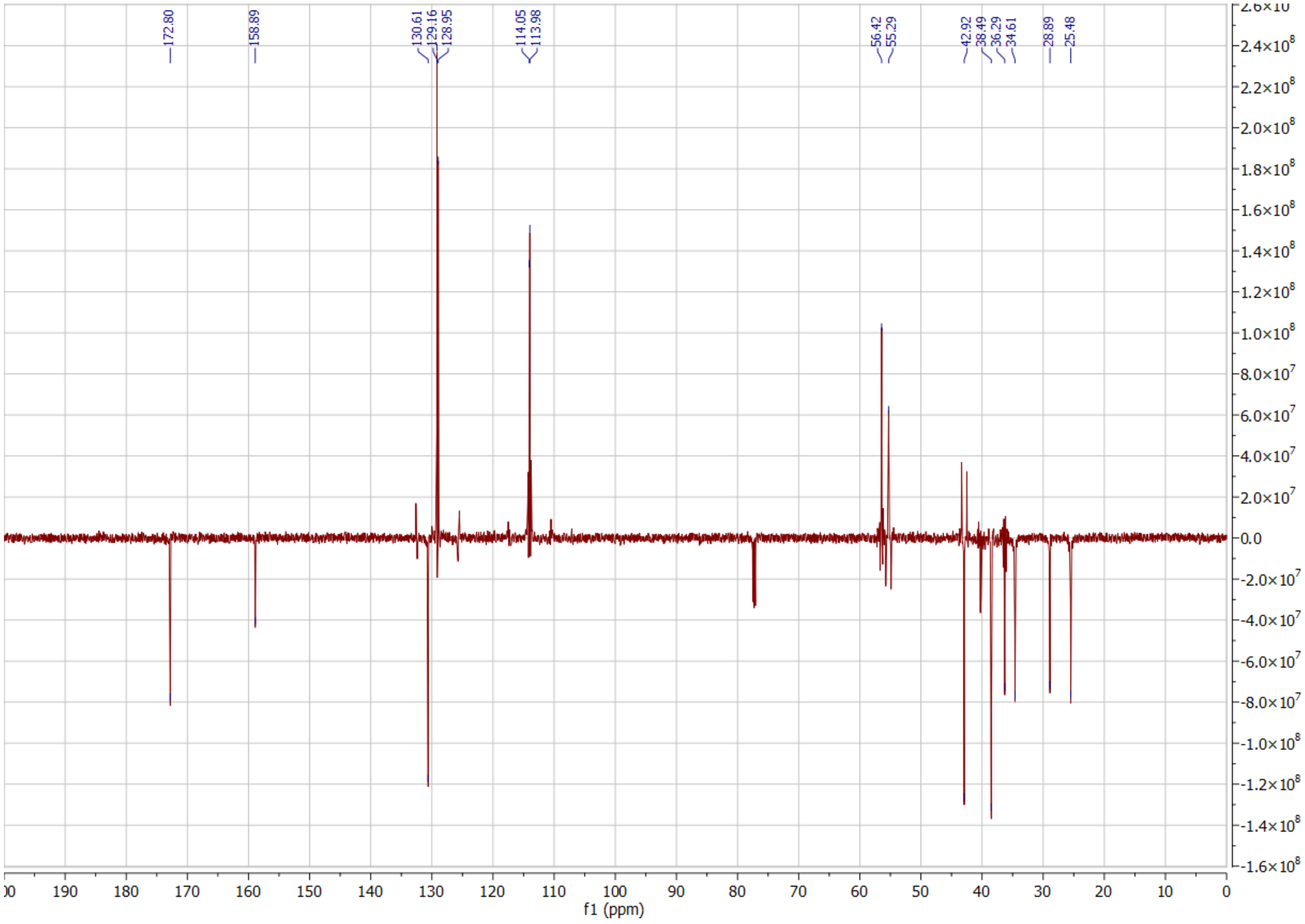

172.80
158.89
130.61
129.16
128.95
114.05
113.98
56.42
55.29
42.92
38.49
36.29
34.61
28.89
25.48
f1 (ppm)

Compound **7**

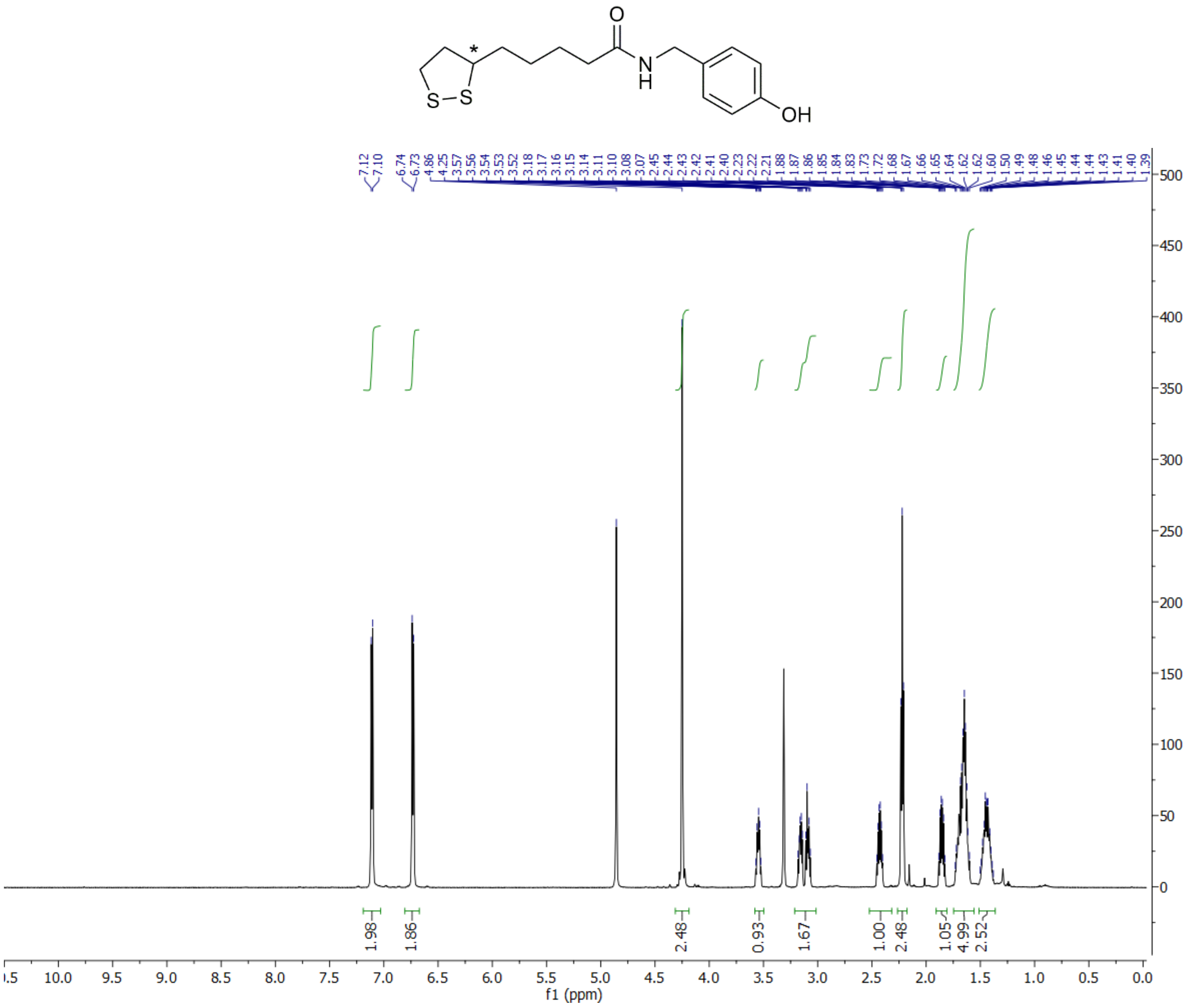

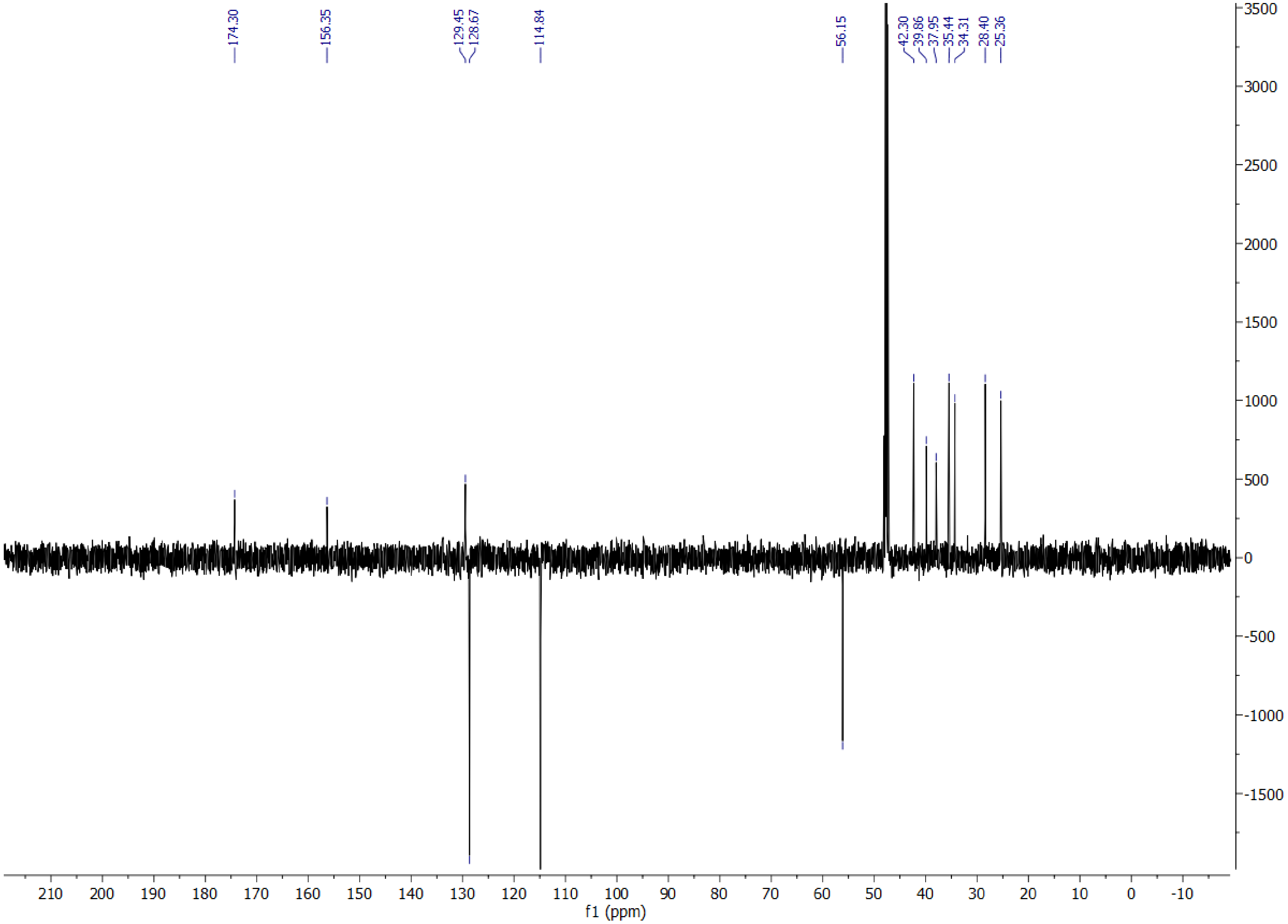

174.30
156.35
129.45
128.67
114.84
56.15
42.30
39.86
37.95
35.44
34.31
28.40
25.36
f1 (ppm)

Compound **8**

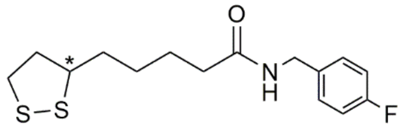

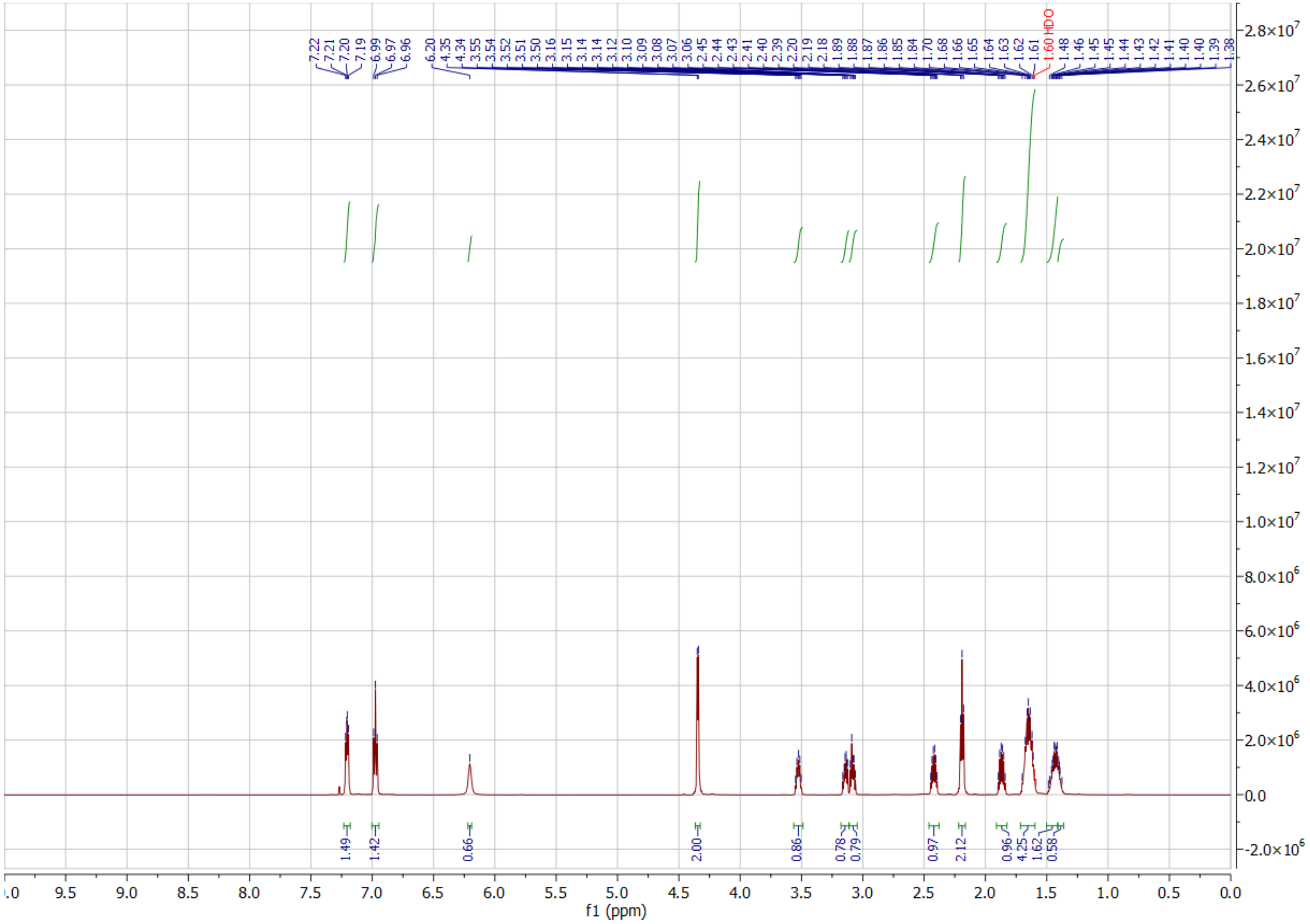

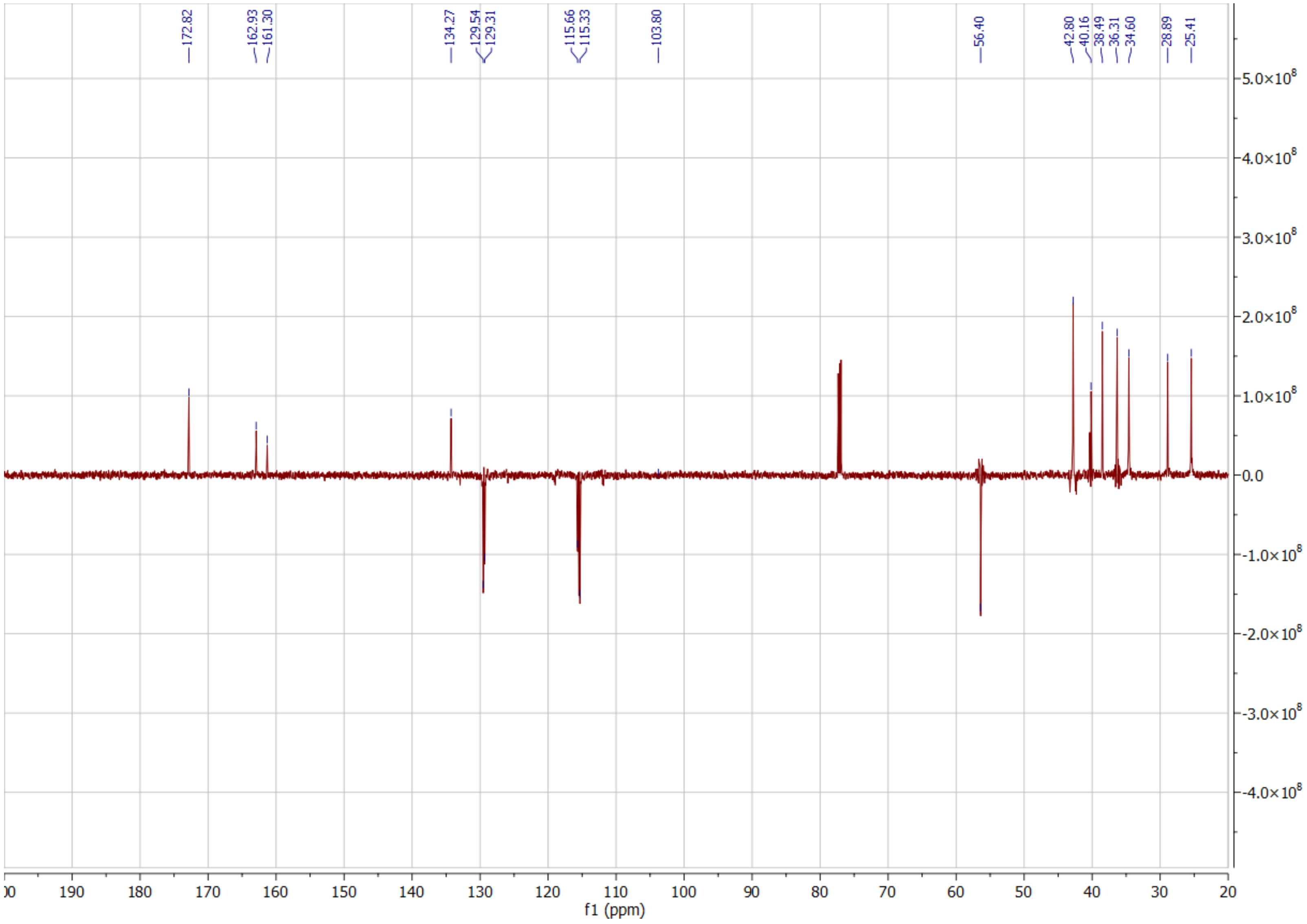
172.82
162.93
161.30
134.27
129.54
129.31
115.66
115.33
103.80
56.40
42.80
40.16
38.49
36.31
34.60
28.89
25.41
5.0×10^8
4.0×10^8
3.0×10^8
2.0×10^8
1.0×10^8
0.0
-1.0×10^8
-2.0×10^8
-3.0×10^8
-4.0×10^8
190 180 170 160 150 140 130 120 110 100 90 80 70 60 50 40 30 20
f1 (ppm)

Compound **9**

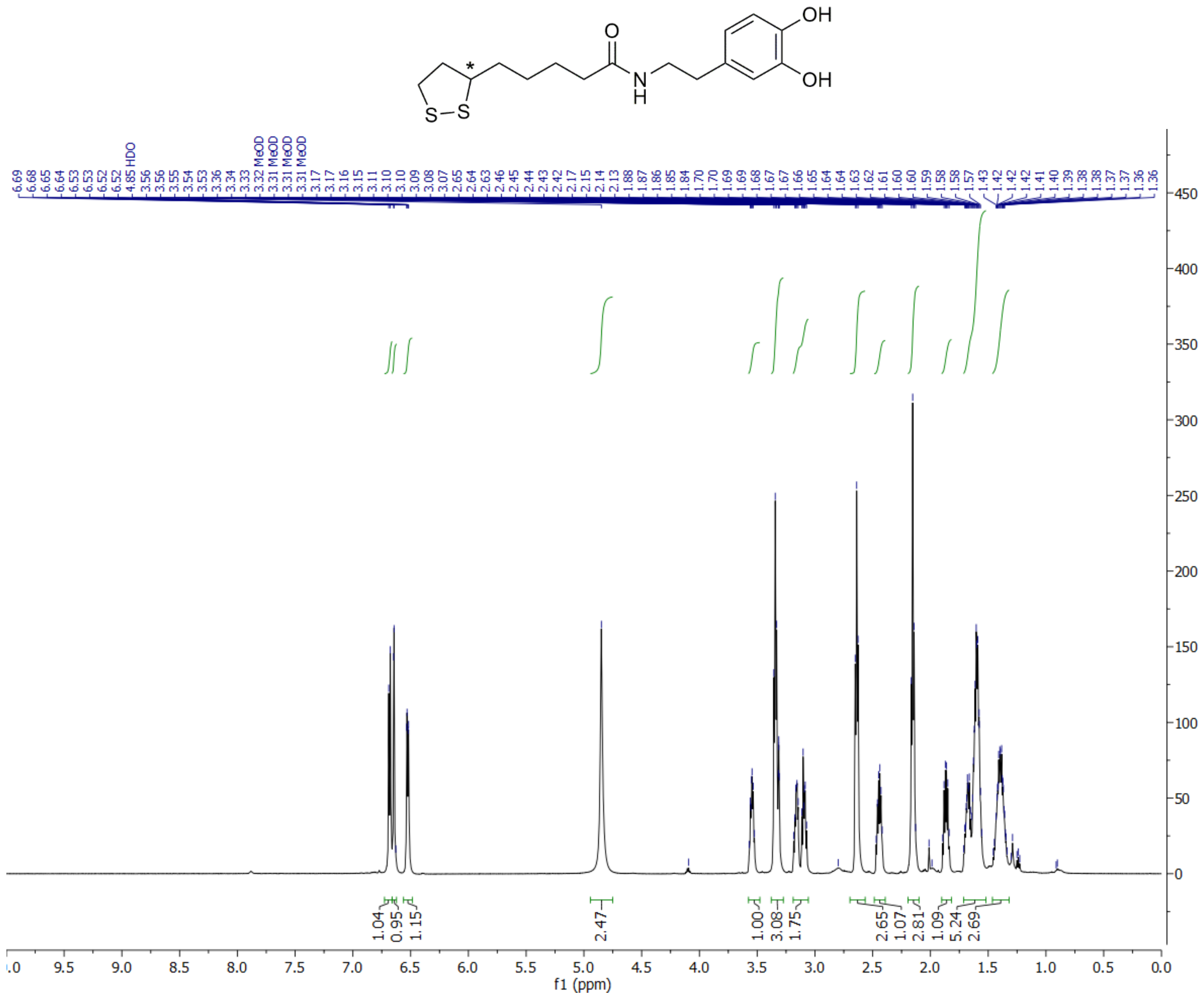

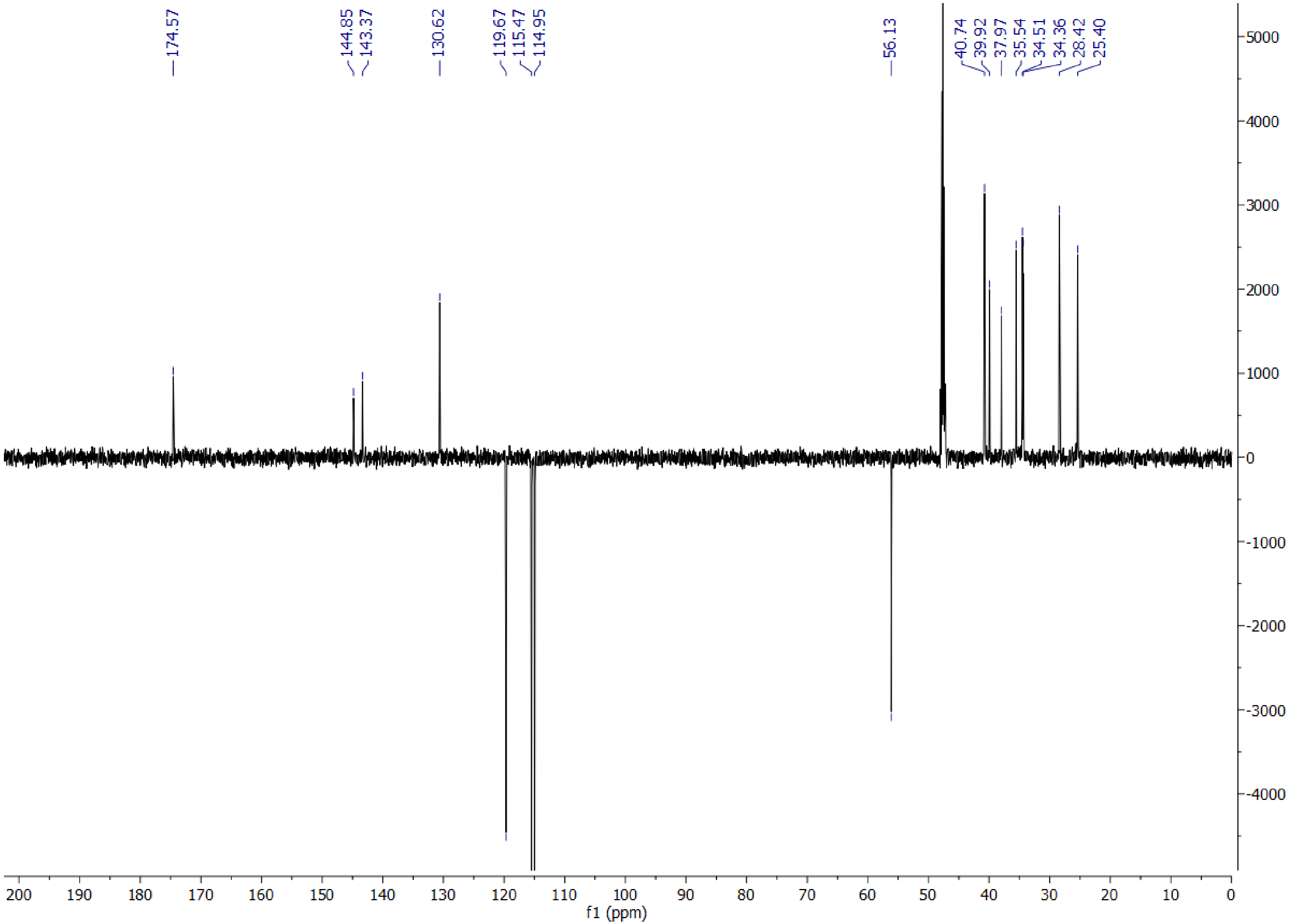
174.57
144.85
143.37
130.62
119.67
115.47
114.95
56.13
40.74
39.92
37.97
35.54
34.51
34.36
28.42
25.40
5000
4000
3000
2000
1000
0
-1000
-2000
-3000
-4000
200 190 180 170 160 150 140 130 120 110 100 90 80 70 60 50 40 30 20 10 0
f1 (ppm)

Figure S20. Scavenger activity of capsaicin through the DPPH assay using EPR spectroscopy

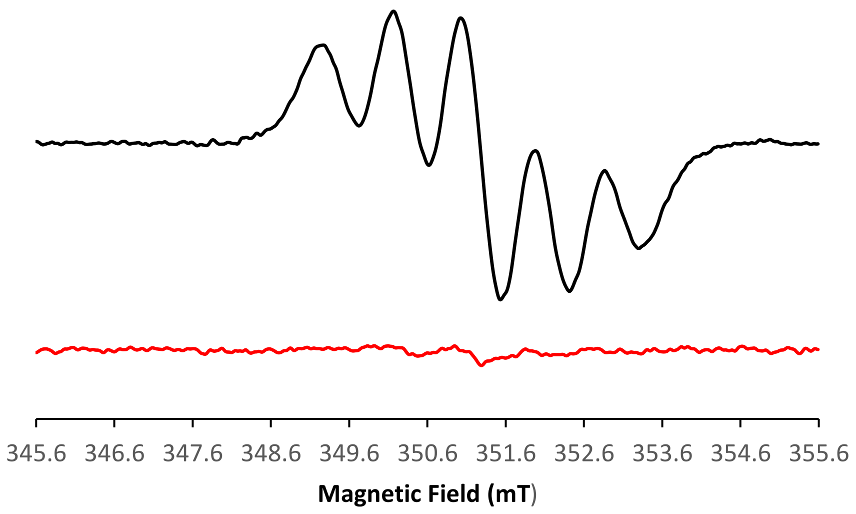

Room temperature (298K) EPR spectra at X-band of DPPH radical before (black line) and after the addition of capsaicin (red line) with 20 minutes of incubation. Experimental conditions: ν=9.869GHz microwave frequency, 2mW microwave power, 0.1mT modulation amplitude.

The solutions were prepared in acetonitrile with a final concentration of 0.1mM of DPPH and 2.2mM of capsaicin.

Room temperature EPR spectra of DPPH radical before (black line) and after the addition of capsaicin (red line). The EPR spectrum of the radical in the presence of the antioxidant shows a strong reduction of the intensity within 20 minutes of incubation time as performed for the other antioxidants.

The calculated value of the R scavenger ratio percentage is 93% for capsaicin.

Figure S21. Cell viability of capsaicin (caps) and tested lipoic amides 1, 4-5, 7 and 9

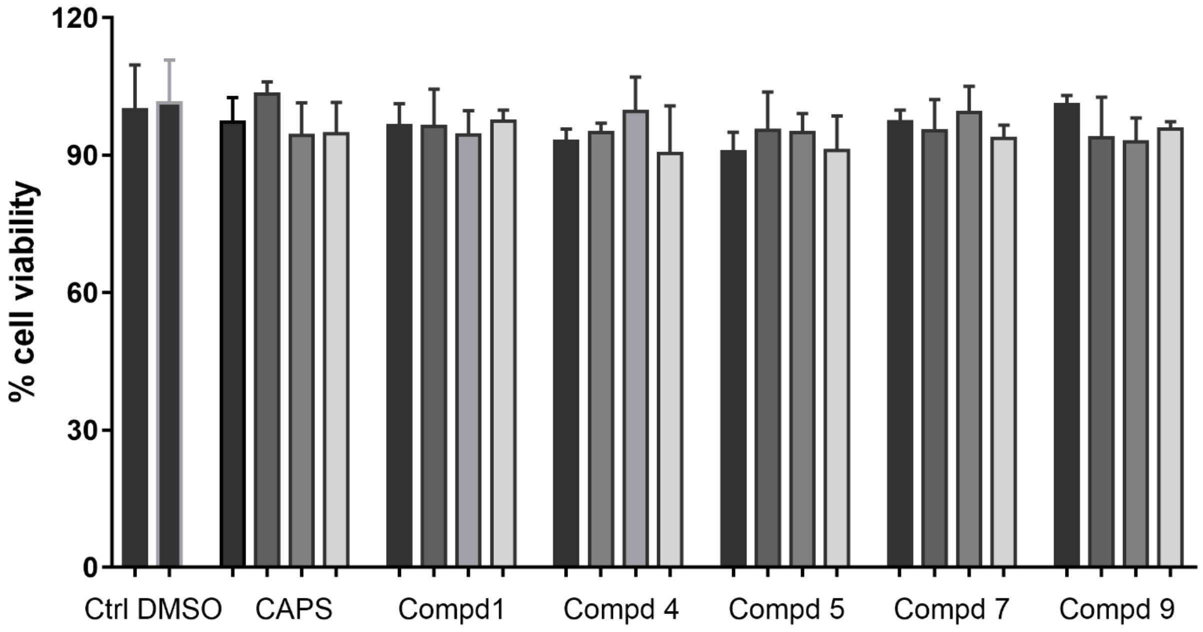

Cell viability expressed as percentage calculated by division of the absorbance values obtained after 48 h of capsaicin or compounds, i.e. **1**, **4-5**, **7** and **9**, incubation at increasing concentration (0.1, 1, 3, 10 μM) by those obtained without substances. Each point represents the mean ± SEM of five independent experiments. No significant differences were measured (ANOVA with post-test).
